# Introducing a sensor to measure budburst and its environmental drivers

**DOI:** 10.3389/fpls.2015.00123

**Published:** 2015-03-10

**Authors:** George J. Kleinknecht, Heather E. Lintz, Anton Kruger, James J. Niemeier, Michael J. Salino-Hugg, Christoph K. Thomas, Christopher J. Still, Youngil Kim

**Affiliations:** ^1^Oregon Climate Change Research Institute, Oregon State UniversityCorvallis, OR, USA; ^2^College of Earth, Ocean, and Atmospheric Sciences, Oregon State University, CorvallisOR, USA; ^3^IIHR-Hydroscience and Engineering, College of Engineering, The University of IowaIowa City, IA, USA; ^4^Department of Micrometeorology, University of BayreuthBayreuth, Germany; ^5^Department of Forest Ecosystems and Society, Oregon State UniversityCorvallis, OR, USA

**Keywords:** budburst, phenology, phenotyping, climate change, temperature, photoperiod, sensing

## Abstract

Budburst is a key adaptive trait that can help us understand how plants respond to a changing climate from the molecular to landscape scale. Despite this, acquisition of budburst data is constrained by a lack of information at the plant scale on the environmental stimuli associated with the release of bud dormancy. Additionally, to date, little effort has been devoted to phenotyping plants in natural populations due to the challenge of accounting for the effect of environmental variation. Nonetheless, natural selection operates on natural populations, and investigation of adaptive phenotypes *in situ* is warranted and can validate results from controlled laboratory experiments. To identify genomic effects on individual plant phenotypes in nature, environmental drivers must be concurrently measured, and characterized. Here, we designed and evaluated a sensor to meet these requirements for temperate woody plants. It was designed for use on a tree branch to measure the timing of budburst together with its key environmental drivers; temperature, and photoperiod. Specifically, we evaluated the sensor through independent corroboration with time-lapse photography and a suite of environmental sampling instruments. We also tested whether the presence of the device on a branch influenced the timing of budburst. Our results indicated the following: the temperatures measured by the budburst sensor’s digital thermometer closely approximated the temperatures measured using a thermocouple touching plant tissue; the photoperiod detector measured ambient light with the same accuracy as did time lapse photography; the budburst sensor accurately detected the timing of budburst; and the sensor itself did not influence the budburst timing of *Populus* clones. Among other potential applications, future use of the sensor may provide plant phenotyping at the landscape level for integration with landscape genomics.

## INTRODUCTION

Budburst is when plants initiate tissue growth from their buds, signaling the end of ecodormancy, and the beginning of the growing season (Lang et al., 1987). The timing of budburst in plants influences biomass accumulation and carbon sequestration, and informs us about the responses of genes and ecosystems to a changing climate (Menzel et al., 2006; Aitken et al., 2008). Climate change may alter the timing of budburst with potentially serious implications (Chuine, 2010), since it could change the amounts of chilling and forcing units sensed by vegetative buds, causing budburst to occur early, late, or not at all (Pope et al., 2013). The advancing date of budburst over recent decades has been documented for a number of species and across a range of biomes (Chuine, 2010). Phenological shifts in agricultural crops can alter the beginning and length of growing seasons, and can also cause crop failures (Chmielewski et al., 2004). A deeper understanding of plant response to climate change is imperative for addressing the effects of future climate change on agriculture and forest management (Badeck et al., 2004).

The timing of vegetative budburst in populations of temperate trees is determined largely by air temperature and genetics (Campbell and Sorenson, 1973; Chuine and Cour, 1999; St. Clair et al., 2005; Harrington et al., 2010). For some species, photoperiod and plant water status provide additional cues to the timing of budburst (Yakovlev et al., 2006; Lagercrantz, 2009; Linares et al., 2012). Air temperature, however, is perhaps the most widely studied and easily measured environmental cue. Phenological models for trees in temperate regions typically include a chilling requirement, representing the effect of cold temperatures on releasing endodormancy (Bailey and Harrington, 2006). We designed a sensor to measure two environmental cues that affect the timing of budburst, temperature and photoperiod, to gather more information about environmental effects on bud phenology in nature.

Despite the increasing evidence for genomic and epigenomic bases of budburst phenology (Yakovlev et al., 2011; Yordanov et al., 2014), our understanding of budburst processes remains limited. Although the molecular basis of budburst is widely studied for model tree species (Hsu et al., 2011; McKown et al., 2013; Yordanov et al., 2014), studies for non-model species, including conifers, are limited to coarse quantitative trait associations (Jermstad et al., 2001), low coverage screens of candidate genes (Eckert et al., 2009), differential gene expressions before and after budburst (Yakovlev et al., 2006, 2008; Mathiason et al., 2009), and some recent work in epigenetics of spruce (Yakovlev et al., 2011). A greater understanding of the molecular and genomic processes behind budburst could be achieved for model and non-model species by measuring the timing of budburst concurrently with its environmental cues.

One challenge we face in deciphering the nature of adaptive processes like budburst in woody plants is the development of new methods of high-throughput phenotyping to relate genes to traits. Molecular data in genomics and systems biology are now being generated in high quantity, which necessitates practical phenotyping methods that can accommodate large numbers of individual plants. Additionally, emulating natural conditions can be difficult in laboratory, green house, or common garden settings (Granier et al., 2006; Poorter et al., 2012). Consequently, it is not surprising to find studies that identify adaptive markers and genes for plants in laboratories that either do not extrapolate to other laboratories (Massonnet et al., 2010) or to natural plant populations (Mishra et al., 2012). Furthermore, to date, there have been very few efforts to identify adaptive genes in natural populations because the environment in most ecosystems is impossible to control, and thus presents a challenge to establish traditional cause-and-effect relationships. These difficulties, in addition to others, hinder advancement of our understanding of the non-linear interactions between a plant’s genes, life stage, growth stage, and the environment (Hänninen and Tanino, 2011).

Data acquisition for the timing of budburst itself is currently limited in scale. Remotely sensed data from satellites are difficult to resolve to a single species, and often budburst occurs during cloudy weather, obscuring the actual date of the event (Schwartz et al., 2002). Human observation data are cost-prohibitive and difficult to collect in remote areas. The eddy covariance method for measuring carbon fluxes can infer aspects of phenology like budburst from increased photosynthetic rates; however, these inferences do not demonstrate cause-and-effect, are not resolvable to individual trees or species, and can be difficult to interpret in sub-optimal weather conditions (Niemand et al., 2005; Chiang and Brown, 2007). Near-surface digital time lapse cameras are not calibrated scientific instruments (Sonnentag et al., 2012); they are not designed to measure temperature at or near buds, and they suffer from issues of battery life, file storage, and impracticality of distribution across a landscape.

Current methods for measuring foliar temperatures also leave room for further innovation. Infrared gas analyzers control leaf temperature to better measure variables like respiration (Bolstad et al., 1999; Xu and Griffin, 2006), but are expensive and are not designed to measure ambient foliar temperatures of organisms in the field. Thermocouples placed against the undersides of needles (Martin et al., 1999) or inside plant tissues (Michaletz and Johnson, 2006) are fragile and risk being moved or broken during long-term deployments. Iteratively calculating leaf temperatures to balance an energy budget (De Boeck et al., 2012) requires collecting other micrometeorological data, which can be an expensive investment in equipment and time. Placing leaves in water baths of known temperatures is useful for assessing heat tolerance, as is the use of freezers to assess cold tolerance (Cunningham and Read, 2006), but these techniques are not suitable for field study. Although thermal cameras are non-invasive and provide greater spatial coverage and temporal sampling than most other approaches (Meron et al., 2013; Prashar and Jones, 2014), they are costly and the image processing is difficult.

Although air temperature alone has proven successful for many phenological models, we know that leaf, bud, and meristem temperatures differ from air temperatures (Grace, 2006; Michaletz and Johnson, 2006), even in moderate environments (Savvides et al., 2013), and that foliar temperatures are a likely more physiologically relevant (Still et al., 2014). The digital thermometer we describe here is durable, ready for long-term field deployment, and designed to provide an indirect metric of foliar temperature. To our knowledge, it is the only existing thermometer designed with a clear acrylic coating to more closely approximate foliar temperature compared to air temperature for cost-effective yet biologically relevant information. The highly localized collection of temperature data provides additional value, since temperatures can vary up to 10°C within a single tree (Stockfors, 2000; Leuzinger and Körner, 2007).

Here, we report results from several sensor validation experiments. Our goal was to innovate sensor technology and validate its performance to improve our capacity to measure budburst and related environmental drivers for high-throughput phenotyping in nature. To ensure the data collected by this sensor are accurate, reliable, and non-invasive, we asked the following questions:

• Does the sensor accurately record temperature, photoperiod, and the timing of budburst?
• Does the presence of the sensor itself influence the timing of budburst?

## MATERIALS AND METHODS

### THE BUDBURST SENSOR (SENSOR)

#### Principle of sensor operation

**FIGURE 1:**
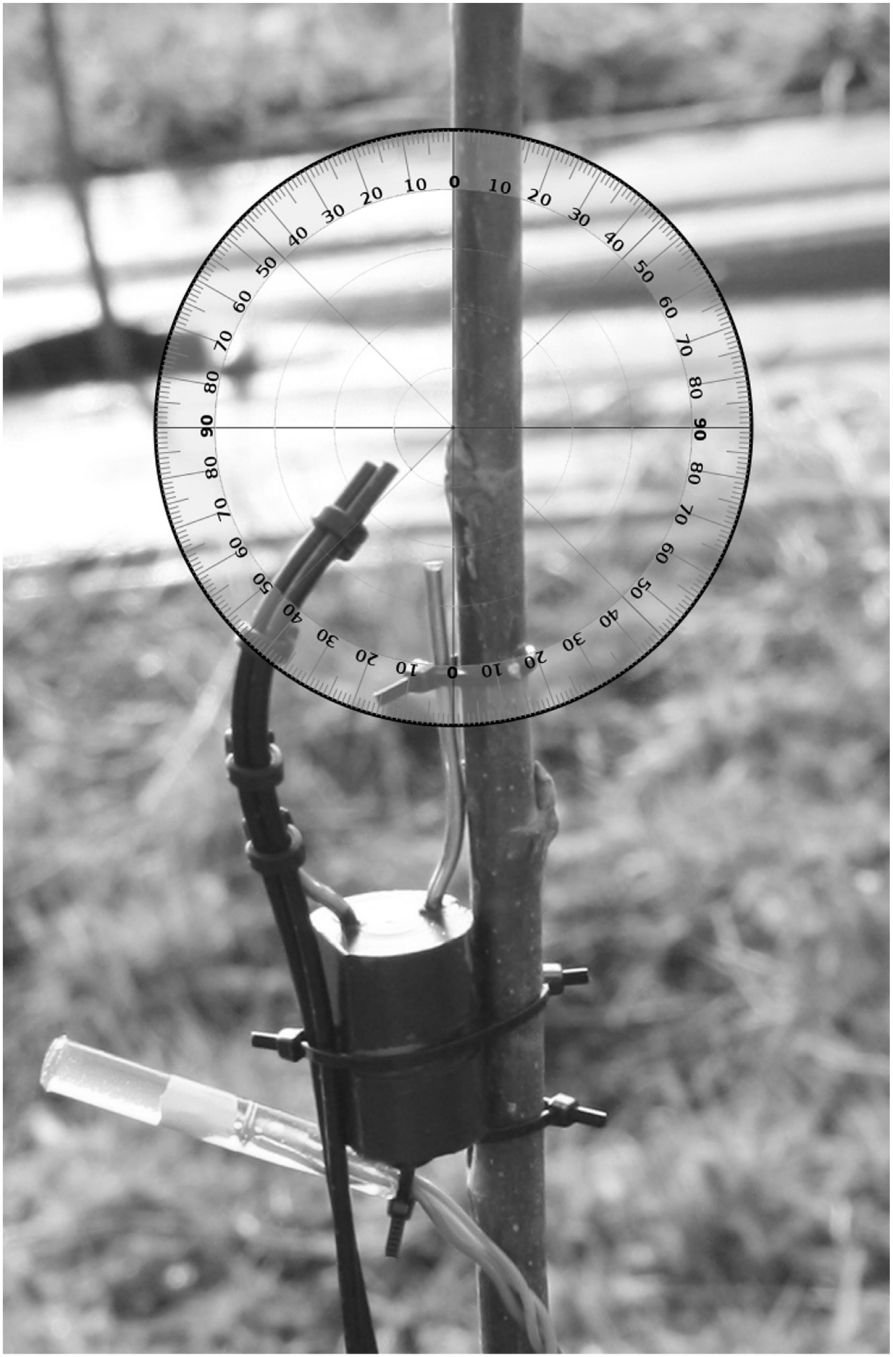
**The bud break sensor’s fiber optic cables targeting a dormant bud, with digital thermometer below**.

**FIGURE 2:**
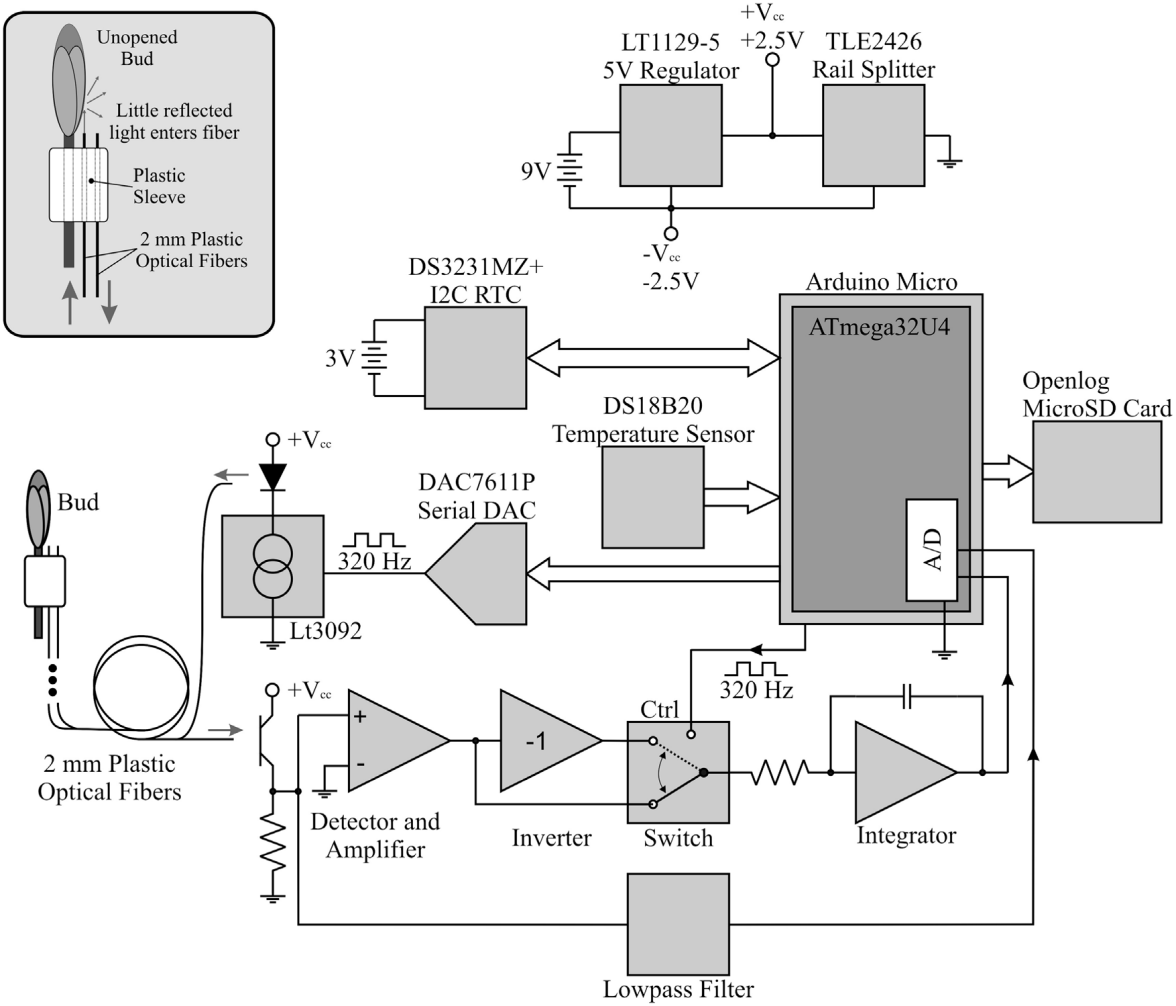
**Schematic diagram of the bud break sensor**.

The budburst sensor used a pair of plastic optical fibers to detect budburst. After being attached to a branch below a bud, one of these fibers guided light from a green LED outward to illuminate the bud while the other fiber received light reflected from the bud and guided the signal to a photodetector and signal amplifier (**Figure 1**). Light pulses were emitted from the illuminating fiber at 320 Hz, a frequency not harmonically related to common man-made light sources, for approximately 2 s (Li et al., 2013). An analog switch routed the light received from the LED through amplifiers with gain +1 (LED illuminated) and -1 (LED dark). This formed the multiplier action of a lock-in detection scheme to rectify the signal (Horowitz and Hill, 1989; Sydenham and Thorn, 2005; Li et al., 2013). Additionally, by averaging the photodetector output when the LED is dark, the sensor provided ambient light information which we used to determine photoperiod. The data were then transmitted to a flash drive and stored with a timestamp (**Figure 2**). The sensor’s integrated circuit thermometer measured kinetic temperatures, and was protected by a coating of clear, waterproof acrylic (**Figure 1**). The electronics and batteries were housed in a plastic case designed to be impervious to water.

#### Using the sensor

The sensor was designed to be simple to use. By connecting the sensor to a terminal emulator on a computer via USB port, one can set the real-time clock, sampling intervals, device identification, and instrument calibration. Sampling intervals can range from once every ten minutes to once per 6 h, allowing users to balance battery life with sampling resolution. During calibration, the sensor’s LED brightens and dims until the device finds a proper signal to noise ratio for the target object. This calibration can be done in the field by pressing a single button, once the sensor is in place and targeting an object of interest. After disconnecting the sensor from the computer and shutting the housing, it is ready for deployment. The main body of the sensor can be strapped to trunks or branches greater than 3 cm diameter, and the wire-mounting system attaches just below the bud with cable (zip) ties. The final positioning of the sensor wires can then be adjusted by moving the bent aluminum wire on the plastic attachment system (**Figure 1**). The sensor needs to be removed after budburst occurs to prevents girdling the tree stem as it begins seasonal growth.

### VALIDATION EXPERIMENTS

#### Temperature

To assess the sensor’s digital thermometer, comparisons were made from two data collection periods. The digital thermometer was compared with air and thermocouple temperatures during the winter, and compared with air, thermocouple, and foliar temperatures during the spring for different trees. Thermocouples and the sensors’ digital thermometers were nestled on test buds. The instruments used for these validation experiments provided a suite of co-occurring measurements to corroborate the digital thermometer’s temperature measurements. Each experiment consisted of sensors, Type-T thermocouples, and one thermal hygrometer (Campbell Scientific® HMP45C) aspirated as per Thomas and Smoot (2013). The spring experimental period also included one thermal imager (FLIR® SC305), a net radiometer (Hukseflux®; NR-01), and a three-dimensional sonic anemometer (Campbell Scientific®; CSAT3). The first test evaluated the digital thermometer’s performance on a potted *Pinus pinea* tree (0.5 m in height) during warm spring weather at Oregon State University’s Botany and Plant Pathology farm just east of Corvallis, Oregon, for 7 days in April and May 2013. The thermal imager was situated 0.5 m above the ground and 2 m away from the plant foliage, pointing 20° east of north to encompass the entire tree. The second test investigated the digital thermometer’s measurements on a taller *Pseudotsuga menziesii* tree during winter conditions on the Oregon State University campus for 14 days in January 2014. Due to the cost of instrumentation, complete replication of the spring data collection period was not possible for the winter experiment.

#### Sensor effect on budburst

Sensor placement onto plant tissue may influence the timing of budburst. This could occur due to changes in the microclimate immediately surrounding a bud if the sensor collects heat energy from the sun during the daytime, or produces its own heat from turning the energy stored in its batteries to electricity. Additionally, thigmotropic responses in plants can occur from moderate, subtle stimuli (Chehab et al., 2009), raising the question of whether budburst may be affected by the sensor touching branches. Furthermore, since conifers can have systemic responses to localized perturbations (Bonello and Blodgett, 2003), a thigmotropic response from a specific bud may influence the entire tree. To address this, we tested whether the presence of the sensor could affect budburst on trees with sensors attached to their branches. Specifically, we tested for a difference in median budburst and a difference in days between phenological phases, with test units consisting of 18 trees with sensors present versus 18 trees with sensors absent. We examined the timing of budburst on 36 trees in a common garden north of Corvallis, Oregon, during March, and April 2014. The trees tested were 2 years old, with trees spaced 1.5 m apart within rows and 3 m apart between rows, and a maximum distance of 49 m between sampled trees. To reduce the influence of genetic variation, all trees studied were clones of the *Populus tremula* ×*Populus alba* hybrid genotype 717-1B4.

Twenty budburst sensors were placed on 16 poplars at the common garden study site on March 1, and the condition and temperature of the buds were monitored for 2 months. Twelve trees had one sensor, four trees had two sensors, and of the 16 trees with no sensors, four had two buds included in the study. This allowed us to test whether phenological differences between control and sensor trees could be due to random chance alone. One vegetative bud per tree was examined on all pairs. Bud condition was recorded during these site visits using five discrete classes (**Table 1**) simplified from Turok et al. (1996). The time-lapse images were compared with sensor output to aid in interpreting the effect of bud swelling and shoot elongation on the sensor output.

**Table 1:** The five discrete budburst classes used, modified from Turok et al. (1996).

0	Dormant bud; no sign of any activity; brown color.
1	Bud swollen; scales reddish; no breakage of bud tissue.
2	Tip of bud is bursting; shoot is visible.
3	Budburst; shoot is green; very young leaves observed.
4	Green leaves separated and growing; venation observable.

#### Photoperiod detector

Sensor photoperiod measurements were corroborated with time lapse cameras (Wingscapes®; TimelapseCam 8.0) during January 2014 on the Oregon State University campus and during April 2014 in a common garden north of Corvallis, Oregon. The cameras operated from 4 am to 9 pm during each sampling event, which provided several hours of dark images before sunrise and after sunset. Photoperiod calculated from brightness values (BVs) of time lapse image pixels was compared to values from the sensor with simple Wilcoxon signed-rank tests.

### DATA ANALYSIS

#### Temperature

We took several steps to process the thermal infrared (TIR) images after the time-lapse image regions of interest (ROIs) containing budburst sensors and thermocouples were identified (12 × 12 pixels in size). After calculating means for the thermal ROI’s raw emittances, the data were radiometrically calibrated in MATLAB (MATLAB and Statistics Toolbox Release 2012b, The MathWorks, Inc., Natick, MA, USA) to correct for emissivity effects in accordance with the Stefan–Boltzmann Law. The thermal images were then corrected for reflected sky temperatures from the foliage surface, as defined by Kirchhoff’s Law. Emissivity values for the foliage were calculated from the Moderate Resolution Imaging Spectroradiometer (MODIS) emissivity libraries for new and old pine needles (MODIS UCSB Emissivity Libraries, n.d.). The emissivity values within these libraries that fell within the thermal imager’s spectral bands were averaged to define our target’s emissivity. We assumed our study trees’ transmissivity to be zero. After the data processing was completed, root mean squared errors (RMSEs) and correlation coefficients were calculated pair-wise between the temperature recorded by the sensor and the calculated foliar temperatures, air temperatures, and thermocouple temperatures. This was performed on the whole time series, as well as smaller components representing day, night, clear, and overcast conditions. Mean temperatures for each hour of the day were also calculated for the budburst sensor’s digital thermometer, air temperature, and foliar temperature. To better assess the biological relevance of the difference between these sensors, chilling and forcing units were calculated for each instrument as per Harrington et al. (2010).

#### Budburst sensing

The time series produced by the sensors were smoothed using a zero-phase fifth-order Butterworth filter to eliminate the diurnal noise in the signal. The time series were then visually compared with time lapse movies of buds opening and visual observations of budburst phase.

#### Photoperiod

Threshold values were used to define daytime periods for BVs from the time lapse images and for digital numbers (DNs) from the budburst sensors’ DNs. Visual examination of the data indicated that sensible threshold values for the budburst sensors ranged from 40 to 70 DNs, while a BV of 20 discriminated light-versus-dark for the time lapse camera. Daytime occurrence was determined using hourly values that exceeded threshold values. Correlation coefficients were then calculated from sensor DN and camera BV data collected during the winter temperature experiment and the spring budburst experiment.

#### Sensor effect on phenology

The visually scored budburst classes were transformed to first differences = *y*(*t*)-*y*(*t*-1), where *y*(*t*) is the bud score at time *t*. The first differences produced the number of days between any given bud score, which we used to develop statistics for the two study test and control groups. We used a Wilcoxon signed-rank test to assess whether the number of days between budburst classes 2 and 3 differed between test and control groups. We also used the same signed-rank test to determine whether there was a statistically significant difference in the date of budburst between sample groups because the signed-rank test was robust to non-normally distributed data (Ott and Longnecker, 2010). For the eight trees with two study buds each, one bud per tree was randomly selected for inclusion in the analysis to maintain independence among analyzed sample units.

## RESULTS

### TEMPERATURE

**FIGURE 3:**
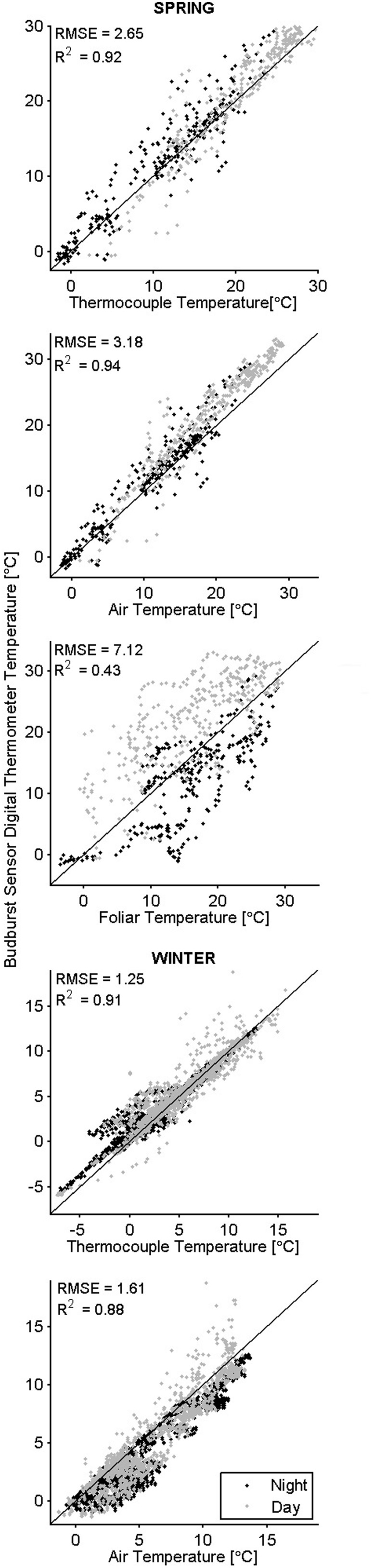
**Co-occurring temperatures color -coded by time of day.** The black 1:1 line indicates perfect agreement between the budburst sensor digital thermometer (*y*-axis) and other measured temperatures (*x*-axis).

**FIGURE 4:**
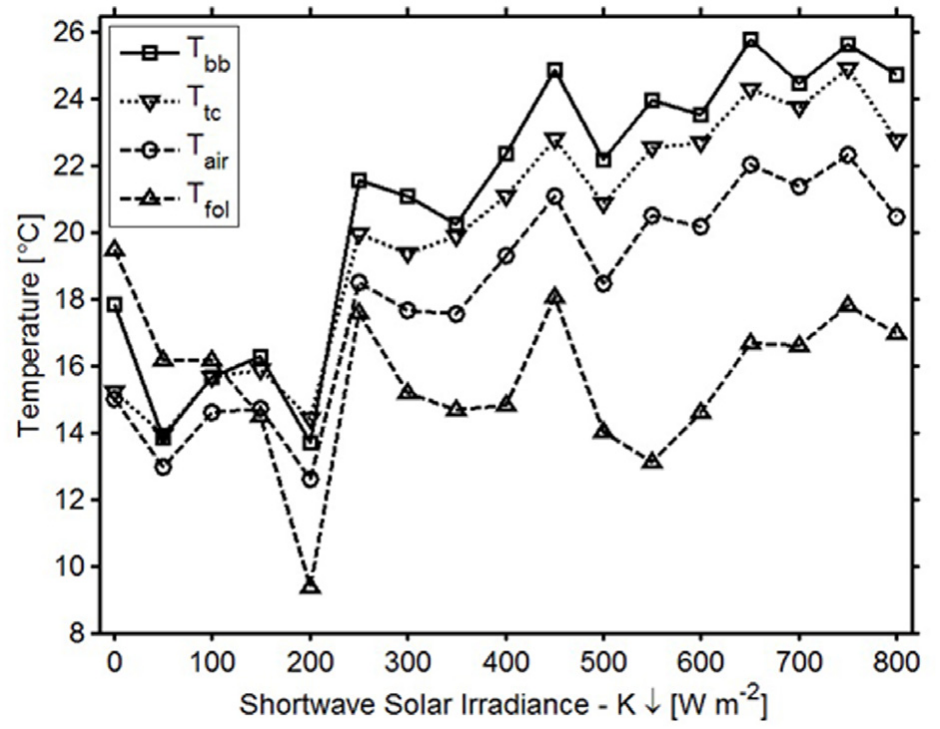
**Average temperatures and corresponding irradiance variations for the bud break sensor’s digital thermometer (squares), air temperature (circles), thermocouple (squares), and foliar temperature (triangles)**.

In general, the temperatures measured by the sensor’s digital thermometer were a better approximation of thermocouple temperatures than were the air and foliar temperatures (**Figure 3**). In the spring when downwelling shortwave radiation was below 150 W m^-2^ (over 12 h a day), digital thermometer values fell between air temperatures measured with the thermohygrometer and TIR foliar temperatures; during higher irradiance conditions, the digital thermometers measured values higher than the air and TIR foliar temperatures (**Figure 4**). In cold weather, chilling units calculated from the digital thermometer were similar to units calculated from other devices, and the same finding held for forcing units calculated in warm weather. Across seasons, the temperatures from the sensor’s digital thermometer best approximated temperatures from a thermocouple, and air temperature secondarily.

Calculations of chilling and forcing units helped to illustrate biological relevance to the differences between measurement types. During the winter data collection period, the chilling units estimated between the measured air temperatures, digital thermometer, and thermocouple were within 31 units of each other, but the differences in accrued forcing units were much larger. Conversely, during the spring data collection period, the forcing units estimated by the measured air and foliar temperatures, digital thermometer, and thermocouple were within 24 units of each other, but differences in accrued chilling units were much larger (**Table 2**).

**Table 2:** Chilling and forcing units calculated from different temperatures (Harrington et al., 2010).

	Time period	Chilling units	Forcing units
Budburst sensor digital thermometer	Winter	1269	183
	Spring	111	505
Thermocouple	Winter	1238	200
	Spring	117	501
Air	Winter	1265	284
	Spring	137	484
Foliar	Spring	128	481

### BUDBURST SENSING

**FIGURE 5:**
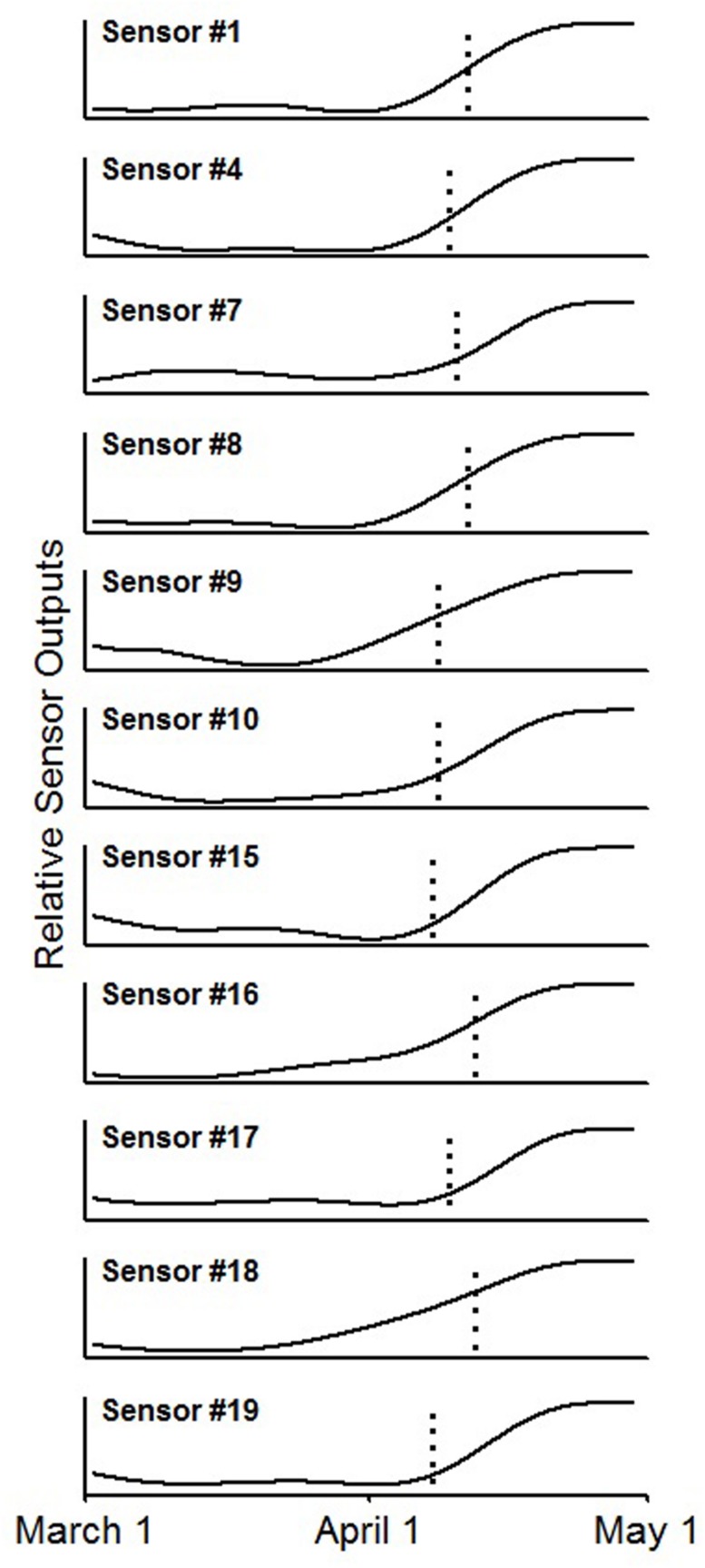
**Smoothed time series from the 11 sensor outputs showing an increased signal near the date of bud break, indicated by the vertical dotted line, using a zero-phase fifth order Butterworth filter**.

By comparing sensor output with confirmed budburst dates derived from site checks and time lapse images, eleven of the 20 sensors (55%) detected budburst (**Figure 5**). For each of these time series, there was a noticeable and abrupt increase in reflected light for the time period measured. The reflected light changed from a low, flat line before budburst to a higher, flat line after budburst. Seven of the failing sensors (35%) succumbed to water damage prior to budburst, and thus, were unable to detect budburst. One sensor’s (5%) signal did not show an increased signal at budburst, and an additional bud did not burst, providing a signal for the sensor (5%) to detect. Overall, of the 12 operational sensors, 91.6% of them successfully detected budburst.

### PHOTOPERIOD

**FIGURE 6:**
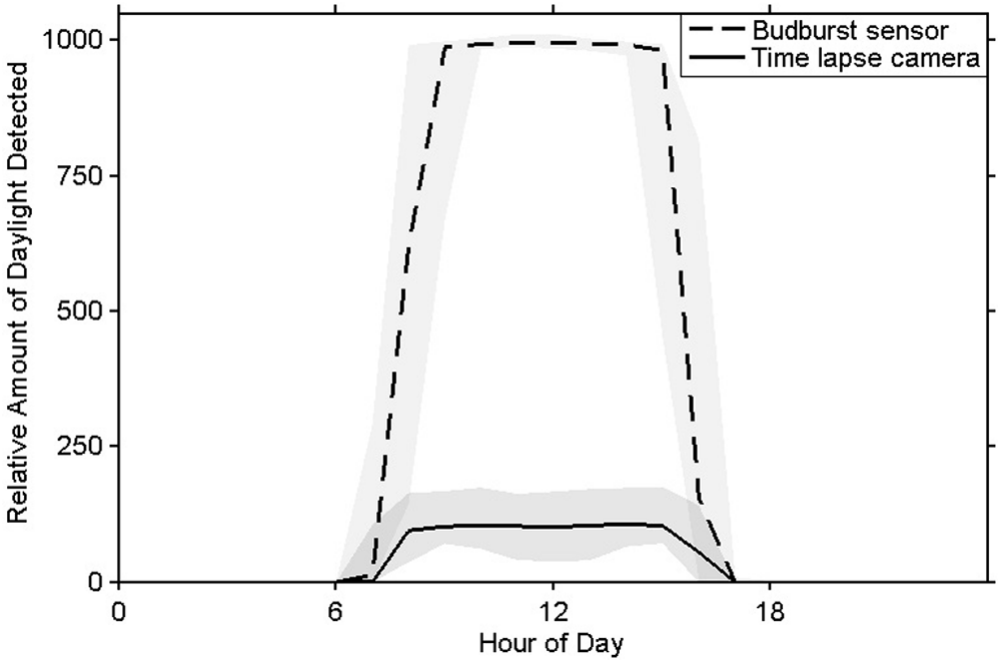
**Mean measurements of daylight from the bud break sensor’s photoperiod detector (in DN units) and time lapse images (in brightness values units) with 5 and 95% quantiles shaded**.

The sensor’s photoperiod detector was able to determine ambient light conditions as could the time lapse imagery (**Figure 6**). Each sensor’s photoperiod values correlated strongly with those calculated from the imagery (*r^2^_winter_* > 0.99, *r^2^_spring_* > 0.98).

### SENSOR EFFECT ON PHENOLOGY

There was no significant difference between median budburst dates for buds with sensors versus buds without sensors (*p* = 0.82). There was also no significant difference between the number of days between budburst classes 2 and 3 amongst control and test groups (*p* = 0.24).

## DISCUSSION

Overall, for the sensors unaffected by water damage, the field tests of each sensing component matched or exceeded our expectations. The signal from the photoperiod detector detected the beginnings and endings of daylight. The acrylic-coated digital thermometer measured temperatures very similarly to thermocouples placed against foliage, and the digital thermometer’s values fell between air and TIR foliar temperatures when solar irradiance was low. The sensor did not influence the timing of budburst, and successfully detected the timing of budburst. These results provide evidence that the budburst sensor is a more versatile choice for measuring budburst than time-lapse cameras because it also collects temperature data.

### TEMPERATURE

The digital thermometer’s durability, ease of deployment, and co-occurring measurements make it a competitive alternative to other temperature measurement devices for field and laboratory research on plants. The close correspondence of measurements between thermocouples and the digital thermometer suggest that the digital thermometer could be used in lieu of thermocouples, which can be fragile and difficult to deploy. When four sensors were deployed on the limbs of a large conifer tree and paired with thermocouples, all of the thermometers were still functioning at the end of data collection, whereas three of the four thermocouples had broken. The self-contained setup also made deployment within a tree crown much quicker for the sensor than for thermocouples, as it does not require external wiring to an energy source and data logger. Thermal cameras can cost 10s of 1000s of dollars and require extensive processing to record accurate temperatures. The need for a constant power source and computer complicates the deployment of thermal cameras in the field. The power source issue extends to air temperature data logging setups as well. Furthermore, the highly localized measurements made by the digital thermometer near a bud provide useful information about the conditions near the bud site, even if it is not measuring bud temperature.

In cold weather, there was better agreement between measurement methods for chilling units than for forcing units (**Table 2**). In warm weather, there was better agreement between calculated forcing units than chilling units. We conclude that chilling and forcing units calculated from data collected by the sensor’s digital thermometer tend to approximate units calculated from other instruments during relevant periods (chilling units in cold weather, forcing units in warm weather).

There were numerous potential sources of error for the measurements of TIR foliar temperature. Systematic deviation between the sensor’s digital thermometer temperatures and measured TIR foliar temperatures in the spring was likely a result of increased latent heat exchange between the foliage and air from transpiration. Increased solar influence, the small size of the tree, and a larger variance between day and night temperatures could explain the higher measurement error in the spring for comparisons with TIR foliar temperature. Accuracy of the thermal camera is reported to be up to ±2°C. Four assumptions made while recording and processing thermal images could have led to additional error: uniform emissivity across pixels, uniform reflectance across pixels, ignored boundary-layer resistance, and a full transmissivity. In agreement with theory (Jones, 1992), TIR foliar temperatures tended to be warmer than the air temperatures during cold weather, and cooler than the air during warm weather.

### BUDBURST SENSING

The 40% rate of damaged sensors was unusually high compared with our thermal validation experiments, which had a 25% sensor damage rate or lower. We believe that the positioning of the sensor housings during the budburst sensing tests made the electronics vulnerable to several heavy rains that occurred during the data collection period and, thus, increased the rate of water damage to the sensors. The plastic housings for the sensors’ electronics were stored on the ground at the base of the tree because the saplings were not large enough to strap the boxes to their trunks. We suspect that the number of sensors damaged could have been reduced if the devices were either kept off the ground or if they had better waterproofing. Additional waterproofing modifications will be made to the housing in future models.

The sensor is in the final innovation phase, and planned modifications will further optimize the instrument for future commercialization. In addition to improved waterproofing, the photodetector that receives the budburst-sensing signal needs to be altered to reduce its sensitivity to sunlight. This change will improve the precision of the sensor’s detection of the budburst event. The attachment of the fiber optic cables to the plant also requires further testing and refinement. The varying ranges of sensor response may also be the result of millimeter differences in distance between the cables and the bud. Future investigation is necessary to ascertain optimal distances and angles to the bud required for optimal sensor output, as well as testing on differing bud types. The results of such tests could enable new methods of processing the budburst-sensing signal; if the magnitude and ranges of sensor responses were more homogenous, then the date of budburst could potentially be inferred from the smoothed signal output exceeding a threshold value. Refinements to the fiber optic cable attachment will also help to shed light on the sensor’s ability to remain in position, relative to the bud, after three, or more months of data collection.

Our experiment confirmed that the sensor presence did not affect bud break of *Populus* clones for the time period studied. However, to be absolutely certain that the sensors have no effect on *Populus* budburst, testing through longer-term deployment is necessary. For example, the sensor batteries can accommodate placement in the fall and retrieval in the spring after budburst. This test will also confirm how well the sensor wires stay in place through a winter. Proof of successful operation throughout a winter prior to budburst will establish the sensor’s viability for studies necessitating such deployment lengths. Additionally, testing across a variety of species is necessary as the physiological response to sensor placement may vary across species or populations (Braam, 2005).

This sensor was designed to sense budburst at the scale of a single bud, in addition to two environmental drivers of budburst. Since not all buds on woody plants break dormancy at the same time, the bud selected may not represent the mean budburst of a whole tree. If it is possible to model these within-tree variations as a function of temperature, then it could enable use of the sensor on a single bud to approximate the trait at the level of a whole tree.

Since it is difficult to control the environment in forests, we suggest that by accounting for the effect of environmental drivers on phenotypes and extracting genomic samples, one could test new hypotheses regarding the genomic basis of those phenotypes. Our sensor allows for measuring plant phenotype in nature with simultaneous measurements of budburst, temperature, and photoperiod on vegetative buds. The measurement of adaptive traits like budburst and their environmental drivers can allow us to step closer to decoding complex processes like the genomic basis of natural phenotypes and how temperate trees will adapt to changing climate (Houle et al., 2010).

This sensor could enable a new caliber of data and insight that can expand our ability to predict the response of natural and agricultural plant populations to a changing climate. Such data could help provide the precision, throughput, and standardization necessary for genome-wide association studies to find the genes underlying adaptive traits, and to better understand molecular, genomic, and ecological mechanisms of phenology and related processes (Neale and Kremer, 2011). Such ground-based quantification also reduces bias from human observations of phenology. Investigations using the budburst sensor in large-scale analyses could help determine if universal response functions can predict budburst in natural populations for evergreen and deciduous species, or determine the degree of correlation between vegetative and reproductive budburst (Wang et al., 2010). Furthermore, since variation in the date of budburst, if measured precisely and continuously, quantifies biological effects of climatic variation (Keeling et al., 1996; Cleland et al., 2007), the sensor’s data can validate satellite measurements and components of land surface models to understand and predict biogeophysical interactions (Studer et al., 2007). Applied research applications in orchards, vineyards, and other agricultural crops further extend the utility of the budburst sensor.

### PHOTOPERIOD DETECTOR

The sensor’s photoperiod detector has been shown to perform as well as a time-lapse camera does for determining photoperiod. Knowing that microsite light availability in forest understories can be highly heterogeneous (Parent and Messier, 1996), the primary advantage of using this photoperiod detector instead of a time-lapse camera is the improved resolution of having a direct measure of photoperiod at the bud. This could yield subtle differences of the light environment at the bud that would not be learned from time-lapse photography or by calculating the solar track from a geographic coordinate. This information, coupled with localized temperature data, will provide researchers with unprecedented environmental data at the bud scale.

## CONCLUSION

We designed a sensor for measuring budburst and its drivers in temperate woody plants. The device is one of the first instruments to measure an adaptive trait in nature along with two environmental factors that influence the phenotype: temperature and photoperiod. We envision this tool will have interdisciplinary application while facilitating progress in the fields of landscape phenomics and budburst phenology. Future work utilizing many budburst sensors across tree populations at a landscape level could provide new insights into the genomes of both model species and conifers, interactions between vegetation and the atmosphere, and the response of ecosystems to climate change. We will not seek a patent for this technology. We invite interested researchers to contact us to explore avenues of future collaboration.

## Conflict of Interest Statement

The authors declare that the research was conducted in the absence of any commercial or financial relationships that could be construed as a potential conflict of interest.
